# The Use of Preliminary Scientific Evidence in Public Health: A Case Study of XMRV

**DOI:** 10.1371/journal.pmed.1001623

**Published:** 2014-04-08

**Authors:** Kumanan Wilson, Katherine Atkinson, Jennifer Keelan

**Affiliations:** 1 Departments of Medicine and of Epidemiology and Community Medicine, University of Ottawa, Ottawa, Canada; 2 Clinical Epidemiology Program, Ottawa Hospital Research Institute, Ottawa, Canada; 3 Dalla Lana School of Public Health, University of Toronto, Toronto, Canada

## Abstract

Kumanan Wilson and colleagues explain how the rapid response to XMRV as a novel pathogen has highlighted some challenges pertaining to policy-making and editorial responsibilities. The impact on policy and the propagation of the initial scientific information may not cease if the evidence is disproven and retracted from the peer-reviewed literature, which creates a challenge for regulators and scientific journals.

*Please see later in the article for the Editors' Summary*

**Summary Points**

- The rapid response to XMRV as a novel pathogen has highlighted some challenges pertaining to policy making and editorial responsibilities in a policy environment influenced by the precautionary principle.
- Once published, preliminary scientific evidence can result in rapid changes in policy and can undergo widespread dissemination via both the Internet and social media.
- The impact on policy and the propagation of the initial scientific information may not cease if the evidence is disproven and retracted from peer-reviewed journals.
- Regulators should consider the use of frameworks to guide the use of the precautionary principle and a separate, more flexible policy stream for precautionary policies.
- Editors should continue to develop strategies to place preliminary scientific evidence of potential public health relevance in context for the public and for policy makers.

The xenotropic murine leukaemia virus-related virus (XMRV) controversy has made evident novel challenges related to integrating scientific evidence into policy making that concerns the safety of blood products and public health in general. The initial publication of an article in the journal *Science* postulating that XMRV was a causative agent for chronic fatigue syndrome (CFS) set forward a cascade of decisions that resulted in the implementation of blood donor deferral policies targeting individuals with any history of CFS [1]. Despite the subsequent failure to replicate the study and the ultimate retraction of the original paper, many of these policies have not been reversed [1]. The XMRV saga highlights new dilemmas pertaining to the publication of preliminary scientific evidence in matters of public health concern. These challenges have been created by the current precautionary policy-making paradigm, and the impact of the Internet and social media as a mechanism for the rapid transmission of health information.

## XMRV as a Potential Transfusion-Transmissible Infection

The story of how XMRV was first identified as a potential transfusion-transmissible infection and the subsequent rejection of this hypothesis has been well documented [1]–[6]. Briefly, the first evidence that XMRV might be a disease-causing agent was reported in 2006 when XMRV genome sequences were detected in a cohort of American men with localized prostate cancer undergoing radical prostatectomy [7]. Concern over the threat of XMRV escalated in 2009 when a research article in *Science* reported that the virus had been identified in 68% of patients with CFS versus only 4% of healthy controls [8]. This publication raised immediate concerns that XMRV could be the biological cause of CFS and could be transmitted through blood transfusion. After months of scientific investigation into possible transfusion transmissibility and the potential links to both CFS and prostate cancer, a consensus emerged that XMRV was a laboratory artifact, and not a threat to blood recipients [6]. At this point, all initial research publications showing a positive association between XMRV and pathogenesis have been retracted, with or without the agreement of the authors [9]–[11].

## Blood Donor Policies

The theory that individuals with CFS might harbor XMRV and that the virus could be transfusion transmissible prompted some blood operators to take a precautionary approach and implement additional donor deferral strategies to protect against this threat (Table 1).

**Table 1:** Countries and their past and present deferral policies relating to CFS and the effects of XMRV on deferral policies.

Country	Previous Deferral Policy	Change Date	Current Deferral Policy	Reason
Australia	Those with active diagnosis of CFS deferred until symptoms resolve	April 28, 2010	Indefinite for any history of diagnosis of CFS	**Safety of blood products, low supply impact. Will revisit in two years' time ** [**25**]
Canada (Canadian Blood Services)	Those with active or symptomatic diagnoses of CFS deferred	April 7, 2010	Indefinite for any history of diagnosis of CFS	**Safety of blood products, low supply impact, pressure from recipient groups ** [**26**]
Canada (Héma-Québec)	Those with active or symptomatic diagnoses of CFS deferred	None	None	**Formal risk evaluation conducted**
United Kingdom	Those with CFS deferred until recovered	November 1, 2010	Permanent for any history of diagnosis of CFS	**Protect blood donor health ** [**27**]
United States	No previous guidance	December, 2010	AABB Bulletin: Active discouragement from donating for any history of CFS diagnosis	**Protect blood donor health and recipients ** [**28**]
Europe (European Centre for Disease Prevention and Control)	No previous guidance	Risk assessment completed July 2011, no change to policy	None	**Awaiting more evidence ** [**29**]
New Zealand	Those with current diagnosis or who have been diagnosed with CFS within past two years deferred	April 21, 2010	Permanent for any history of diagnosis of CFS	**Align with international policy change ** [**30**]

At present, no countries are screening for XMRV or have banned individuals who test positive for XMRV from donating blood, organs, or other tissues. However, since it remains possible that CFS is caused by a retroviral agent, several countries adopted strong precautionary approaches [12] by introducing indefinite or permanent deferrals for blood donation from donors with any history of CFS diagnosis (e.g., Canadian Blood Services, Australia, United Kingdom). Other jurisdictions (e.g., European Union, Héma-Québec) chose to wait for more evidence to accumulate before making a decision regarding the threat of XMRV.

Unlike other cases where high-risk groups protested being the target of donor deferral, the CFS community embraced the restriction. Rather than stigmatizing CFS sufferers, the identification of a possible infectious basis was seen as validation for a disease often characterized as a type of psychological disorder. The CFS community advocated for the implementation of these additional donor deferral policies as well as for donor education and self-deferrals within their communities [13].

Three years later, despite a thorough evaluation of risk and a series of publications discrediting the threat of XMRV, donor deferral policies still exist that target individuals with any past or present diagnosis of CFS. In many of these jurisdictions, these retained policies are likely a consequence of governance or regulatory processes that make it difficult to reverse policy. Nevertheless, they highlight challenges related to the elimination of policies that were introduced on a precautionary basis. By themselves, the CFS deferral policies are not likely to have a significant impact on the supply of blood products as only a very small portion of blood donors are affected. However, failing to revisit precautionary decisions once new evidence becomes available could result in the accumulation of scientifically unjustified policies. This could contribute to blood shortages by unnecessarily deferring groups of potential blood donors and could lead to self-deferral by potential donors due to the ambiguity of existing policies.

## Science, Social Media, and the Precautionary Principle

XMRV highlights a new dilemma relating to the publication of preliminary evidence in matters of public health concern. This dilemma has been created because of a change in the way public health decisions are being made and how preliminary scientific information is being disseminated. One of the legacies of the transfusion transmission of HIV and hepatitis C was the adoption of precaution as a guiding principle in blood safety decisions and in public health in general [14]. The precautionary principle advocates for the introduction of safety measures based on preliminary scientific evidence [15]. Such an approach was found to be successful for managing the risk of transfusion transmission of variant Creutzfeldt-Jakob disease [16]. However, this approach carries potential dangers as made evident by the XMRV experience. Traditionally, scientific journals published provocative hypothesis-generating evidence, which passed peer review with an expectation that other researchers would seek to replicate the results and ultimately confirm the validity of the findings. In the era of precautionary decision making in public health, preliminary evidence may be acted upon before confirmatory evidence is available.

Further complicating matters, preliminary scientific evidence may be rapidly disseminated via the Internet and, in particular, social media. The Internet and social media have been invaluable in allowing individuals to access information about health, and as a means for scientific evidence to be disseminated. However, this is a double-edged sword. Stakeholder groups may disseminate and distort scientific evidence and the meme may persist even after the refutation of the original evidence. Perhaps the most infamous illustration was the publication of the since withdrawn article postulating a link between the MMR vaccine and autism [17]. Despite numerous studies refuting the association, the rumor continues to exist, largely disseminated by the Internet, and has had a very tangible impact on childhood vaccination rates, which has subsequently contributed to measles outbreaks [18]. The suspected and since rejected link between thimerosal and autism has also been similarly problematic [19]. The Institute of Medicine (IOM) invoked the precautionary principle when arguing for a removal of the preservative [20]. This fueled antivaccination sentiment and, despite the absence of studies supporting the association and a subsequent statement from the IOM articulating that the link did not exist, the impact of the original precautionary statement has persisted.

The XMRV story illustrates challenges with acting on early scientific evidence. Policies implemented under precaution are essential to maintaining public health safety as well as public confidence in the system [16]. In an ideal world this would be a prudent approach under the following conditions: policies are revisited in the face of new scientific evidence, regulatory policies could be rapidly changed, and the initial impact of revoked policies could be combated through effective risk communication. Unfortunately, these conditions often do not exist. In many instances policies are not revisited in a timely manner. Even when they are, the regulatory process can be lengthy and there also may be a reluctance to withdraw policies in the face of new evidence for fear of creating the perception that the public is being put at risk.

## Recommendations

**Figure 1:**
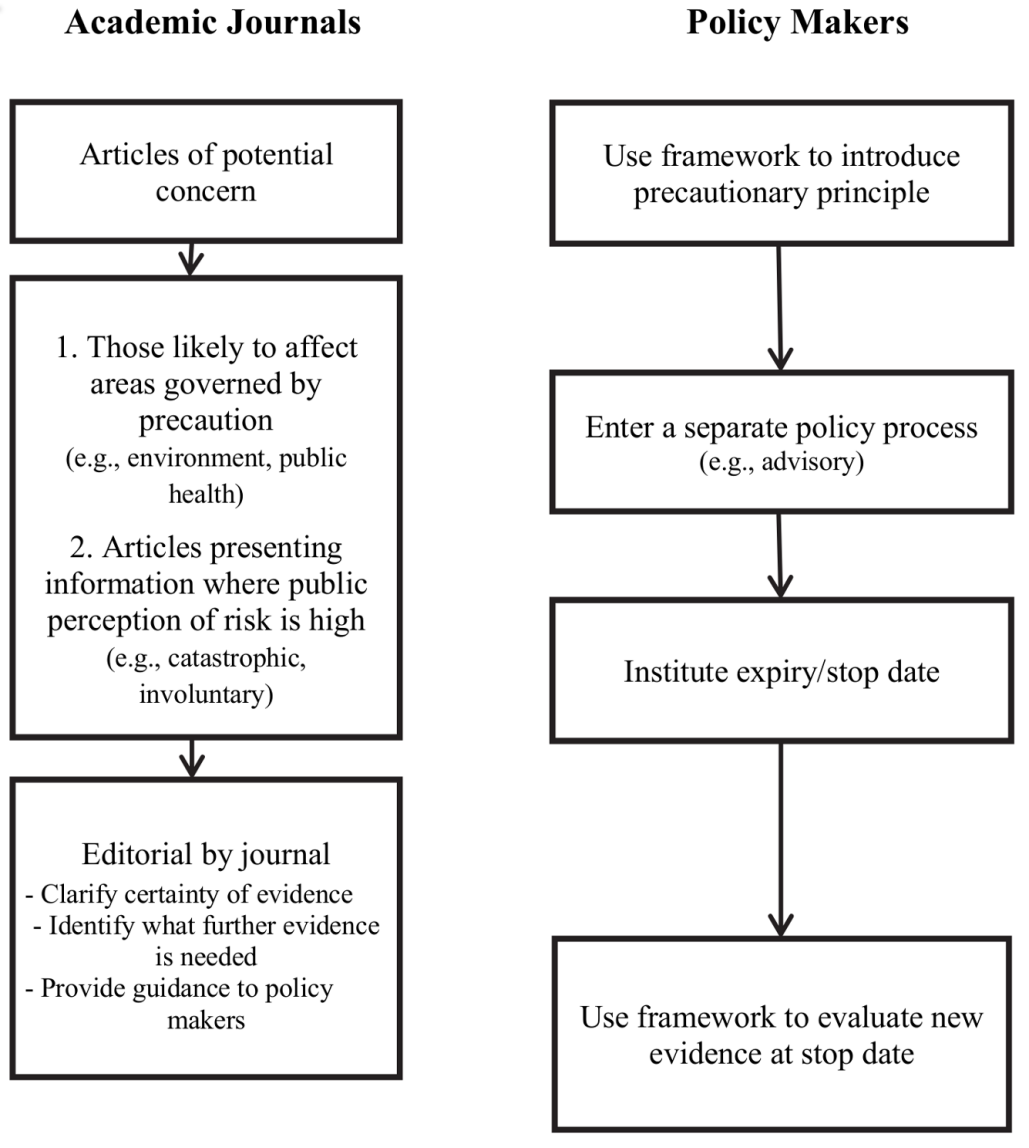
Recommendations for policy makers and journal editors.

The experience with XMRV, like the past experiences with vaccines and autism, illustrates some of the emerging challenges related to the publication of early evidence in areas of public health concern. The primary responsibility for addressing this challenge lies with the regulatory process. However, scientific journals publishing basic research need to take into consideration the dual impact of the dissemination of information via the Internet/social media by advocacy groups and the precautionary principle policy-making paradigm in public health (Figure 1).

### The Regulatory Process

The use of frameworks to guide the initial and ongoing use of the precautionary principle by policy makers is a useful first step to address this challenge [12],[15],[21]. These can help clarify whether an emerging threat is suitable for a precautionary approach depending on the severity of the threat and the consequences of taking action to remove the threat. If a framework had been used, XMRV may not have warranted precautionary action as the condition was not fatal, had arguably limited morbidity, and the virus was potentially not widespread.

Frameworks can also be useful to guide the transitioning of precautionary policies when new evidence becomes available. A fundamental component of the European Union's communication on the precautionary principle is that the policy be “subject to review, in the light of new scientific data” [15]. However, the amount of evidence needed to remove the policy is often much greater than the amount of evidence of risk needed to introduce the policy in the first instance. The length of time to change policies related to donation of blood from men having sex with men in many jurisdictions is illustrative of this dilemma [22],[23].

To overcome this obstacle, we recommend that threats that qualify for the use of precaution are brought through a separate policy process from policy decisions made on more definitive evidence. A distinctive policy process could funnel decisions through a less onerous process in order to be changed when new evidence becomes available. For example, in the instance of XMRV, the American Association of Blood Banks (AABB) chose to issue an advisory to its members recommending that they defer blood donations from individuals with any history of CFS. This approach facilitated the process of revising and ultimately removing the recommendation as more evidence became available. We further recommend that precautionary-based decisions have an expiry/stop date at which the evidence is reviewed and the decision be made into a permanent policy, removed completely, or continued for another set period. Such an approach would guard against the accumulation of measures with limited scientific basis.

### Academic Journals

What steps can scientific journals take to navigate the current public health policy-making environment? Even if the regulatory process is changed to be more responsive to new evidence that negates existing precautionary policies, there is still the risk that the communication of preliminary evidence may have a lasting impact, particularly where there are advocacy groups that support and disseminate the evidence. These new realities, however, should not discourage the publication of provocative novel findings.

Journals have already taken several steps to correct or clarify scientific evidence. Some of these steps have included encouraging authors to add balanced information to papers, permitting post-publication comments on their websites, as well as issuing expressions of concern and retractions through these media. The National Library of Medicine has similarly enabled authors to comment and discuss publications through the creation of PubMed Commons. To supplement these, we would also suggest some further initiatives at and prior to publication for articles of potentially high policy impact and public interest. High policy impact publications include those that present findings relevant to the environment or public health, domains influenced by the precautionary principle. High public interest articles that may be rapidly disseminated among advocacy groups include those that concern areas where the public perception of risk associated with the topic is high, i.e., those addressing risks of an involuntary or catastrophic nature [24]. Some journals commission editorials and commentaries to contextualize research findings that might cause alarm. We recommend wider uptake of this practice. These editorials could clarify both the scientific context of the article's findings and the policy context of the research. In a structured manner, these could state where on the scale of scientific certainty this research falls, which findings are preliminary and require more evidence and which findings are substantive enough to warrant action, and what future research is required to clarify any uncertainty. This is particularly important because standard methods for evaluating evidence would likely not apply as the evidence on which precautionary decisions are being made is often based on a basic science study, a case report, or a case series.

## Conclusion

The potential transfusion transmission of XMRV has highlighted novel challenges pertaining to policy making and the publication of preliminary scientific evidence on matters of public health. In particular, it demonstrates challenges in an era in which decision making concerning public health is increasingly influenced by the precautionary principle, and where scientific findings linked to areas of public interest are rapidly disseminated via the Internet and social media.

## Abbreviations

CFS: chronic fatigue syndrome
XMRV: xenotropic murine leukaemia virus-related virus
